# Impact of Biofilms on Chronic Infections and Medical Challenges

**DOI:** 10.7759/cureus.48204

**Published:** 2023-11-03

**Authors:** Sakshi Mendhe, Ankit Badge, Sarita Ugemuge, Dhurba Chandi

**Affiliations:** 1 Microbiology, Datta Meghe Medical College, Datta Meghe Institute of Higher Education and Research (DU), Nagpur, IND; 2 Microbiology, Jawaharlal Nehru Medical College, Datta Meghe Institute of Higher Education and Research (DU), Wardha, IND

**Keywords:** biofilms, medical devices, prevalence, antibiotic resistance, chronic infections

## Abstract

Biofilms which are intricate colonies of bacteria encapsulated in a self-produced matrix are becoming more widely recognized for their importance in persistent infections. Biofilm-related infections provide distinct diagnostic and therapy issues needing novel approaches. Biofilms are common in clinical settings and contribute to the persistence of diseases related to medical devices, dental health, respiratory disorders, and chronic infection. Overcoming these problems requires a thorough understanding of the elements that influence biofilm development and their complex interactions within the microbial community. Emerging diagnostic techniques and therapy approaches that target biofilm-related disorders at different levels give hope for improved patient outcomes. This review looks at how biofilm formation affects chronic infections in a variety of ways, including increased drug resistance, immune system evasion, and delayed diagnosis.

## Introduction and background

Biofilms are intricate bacterial colonies that grow in various settings, including medical devices, chronic infections, and the human body. Biofilms are noticed by a safety model of extracellular polymeric substances that surround the bacteria and pose an appreciable therapy and diagnostic challenge [1]. Biofilms have different features that distinguish them from free-floating bacteria in the context of persistent infection. These changes have significant therapeutic repercussions, including increased antibiotic resistance, chronic inflammation, tissue damage, and diagnostic challenges [2]. The capacity of bacterial biofilms to confer antibiotic resistance is one of their most significant effects. Bacteria are protected from the effects of antibiotics within the protective biofilm matrix, making them highly resistant to antibiotics [3]. Biofilms also include increased resistance to the immune system and medications. The importance of individual treatment plans and current research projects aiming at understanding and treating the complexities of biofilm-related infection is highlighted by this distinction [4]. Chronic infections that are linked to biofilms are difficult to diagnose. When biofilms are present, conventional diagnostic methods to find bacteria in human fluids or tissues may produce false-negative results. Biofilms have a major role in antibiotic resistance. Biofilm creates a defense that shields bacteria from the effects of antibiotics, reducing the absorption and effectiveness of drugs [5]. Additionally, bacteria found in biofilms frequently grow more slowly and adopt unique metabolic states, making them less vulnerable to antibiotics produced for cells that divide quickly. These difficulties are made even more difficult by the various microenvironments found within biofilms and the possibility of genetic changes that promote antibiotic resistance. Biofilms are frequently seen on medical implants and devices, such as catheters and prosthetic joints, which can cause difficulties in various clinical settings [6]. The chronic infections that are difficult to treat and beyond this, biofilms affect gastrointestinal infections, respiratory infections, chronic infections, urinary tract infections, and chronic otitis media [7]. The objective of this review is to highlight the multifaceted challenges posed by biofilms in chronic infections, including antibiotic resistance, and their impact on diverse medical settings.

## Review

Method

To conduct a review literature search, we used the following databases PubMed, Scopus, and Google Scholar. We searched for articles published between 2020 and 2023. Use the following search terms: (Chronic infection) OR (Infection) AND (antibiotic resistance) OR (Antimicrobial) OR (Multidrug resistance) AND (Medical devices) OR (Prosthetics devices). We applied the following inclusion criteria for the final review. Original research article, English language, peer-reviewed, Full-text available, and published in the specified time frame.

Articles Screened

**Figure 1:**
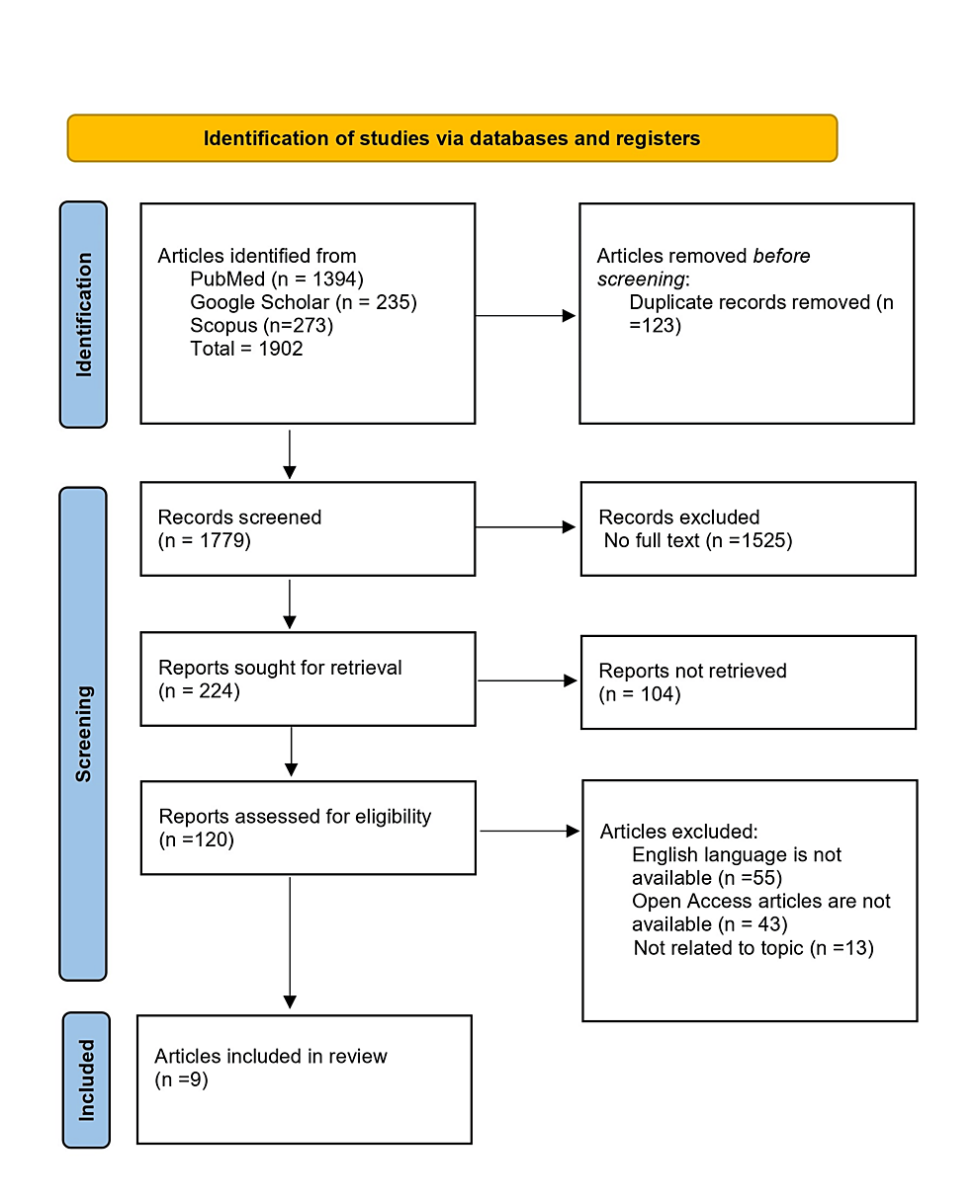
PRISMA flow chart n: Number of studies; PRISMA: Preferred Reporting Items for Systematic Reviews

After conducting the initial screening, we identified 1902 articles across the searched databases. When we excluded duplicates (n=123) and completed an initial screening of the remaining articles, we excluded 1525 articles. After full-text screening of the remaining articles, we excluded 104 articles, we excluded 111 articles for not meeting the inclusion criteria, English language was not available, and open access articles were not available, leaving a total of nine articles for the final review from the years 2020 to 2023 (Figure 1).

Articles included in the review show the impact of biofilms on chronic infections (Table 1).

**Table 1:** Studies included in the review

Sr.no	Author	Year	Conclusion	Findings
1.	Barzegari et al. [8]	2020	Biofilms pose a serious clinical problem and are a major cause of many microbial and chronic infection.	The battle of probiotics and their derivatives against biofilms.
2.	Vestby et al. [4]	2020	It has been proposed that biofilms are connected to an expanding variety of diseases. The bulk of the time, this is predicated on the discovery of biofilm-like formations in patient exudates, autopsies, and biopsies	Bacterial biofilm and its role in the pathogenesis of disease. antibiotics.
3.	Bari et al. [9]	2023	Specifically, the solid structure of biofilms makes drug delivery systems ineffective and reduces the effectiveness of therapy.	Combination drug action for biofilm removal using synthetic and natural agents
4.	Yu et al. [10]	2023	The interaction between airway inflammation and infection in cystic fibrosis has become an important aspect of pathobiology of the condition.	Respiratory infection and inflammation in cystic fibrosis
5.	Ramakrishan et al. [11]	2022	Microbial infection and use of clinical treatments to treat patients, biofilm associated infections continue to pose a serious threat to human health.	Respiratory infection and inflammation in cystic fibrosis
6.	Hibstu et al. [12]	2022	Compatibility with current quality and safety standards; long-term stability of phage preparations; effective assays for phage screening	A different approach to fight bacterial infections
7.	Achinas et al. [13]	2020	The formation of biofilm within bioreactors causes a reduction in the efficiency of the bioreactor and, in some bioreactor applications, causes health issues.	A technological understanding of biofilm detection techniques
8.	Schulze et al. [14]	2021	Biofilms contribute to a number of human diseases and have a high level of antibiotic resistance; bacterial biofilms present a significant challenge to our healthcare system.	Biofilms by bacterial human pathogens: clinical importance- development, composition and rule- therapeutical plan
9.	Zhang et al. [15]	2020	Infections caused by biofilms are still a major worry for healthcare services. High biofilm resistance to existing antibiotic therapy appears to be a significant obstacle in this area.	Promising therapeutic strategies against microbial biofilm challenges

In three studies [9,14,15], the biofilms of human bacterial pathogens have clinical significance, development, composition, therapeutic plan, and drug action combined to remove biofilms with synthetic and natural agents. Two studies [10,11] are the same and assumed about respiratory infection and inflammation in cystic fibrosis. Two studies [4,12] show a bacterial biofilm and its role in the pathogenesis of disease antibiotics. One study [13] evaluates a technological understanding of biofilm detection techniques. One study [8] is about the battle of probiotics and their derivatives against biofilms. 

The complex impact of bacterial biofilms on chronic infections

In chronic infections, bacterial biofilm formation can have substantial clinical effects that differ from infections induced by free-floating bacteria [4]. A sophisticated bacterial community known as a biofilm is contained in an intricate extracellular matrix that it has self-produced. Bacterial biofilm formation in chronic infections is linked to decreased immune system and antibiotic resistance, persistent inflammation and tissue damage, and diagnostic problems. These distinctions from bacterial infections underline the necessity of customized treatment approaches and ongoing research to understand better and combat biofilm-related disorders [4]. Biofilm-associated chronic conditions are also more difficult to diagnose. Traditional diagnostic techniques that detect bacteria in body fluids or tissues may produce false-negative results because biofilms are difficult to detect using standard methods [16].

Biofilm-mediated antibiotic resistance

Bacterial biofilms contribute considerably to antibiotic resistance by building a protective environment that protects organisms from the effects of antibiotics [4]. This matrix limits antibiotic penetration, decreasing the medications' effectiveness in destroying microorganisms within. Furthermore, bacteria in biofilms frequently grow more slowly and in distinct metabolic states, making them less susceptible to antibiotics meant to kill actively dividing cells. Treatment is further complicated by the varied microenvironments found inside biofilms [17]. Furthermore, genetic modifications can occur within biofilms, facilitating antibiotic resistance development [18]. These include using combination therapy with multiple antibiotics to target biofilm bacteria from different angles, using biofilm-disrupting agents to break down the protective matrix, using nanoparticles or drug delivery systems to enhance antibiotic penetration, developing specific anti-biofilm compounds, exploring phage therapy to target biofilm bacteria, using quorum sensing inhibitors to disrupt biofilm formation, and utilizing physical approaches [9].

The prevalence of bacterial biofilms

Bacterial biofilms are common in a variety of clinical settings and their presence has a significant impact on treatment success and patient diagnosis. Biofilms are routinely seen on medical implants and devices which include catheters and prosthetic joints, increasing the risk of persistent and difficult-to-treat infections of Biofilms in dental and oral health contribute to disorders such as dental caries and periodontitis, which can lead to tooth loss and negatively effect on general health [19]. The prevalence of biofilm formation on medical devices is estimated to range from 65% to 80% across various healthcare settings worldwide [20]. Biofilms are frequently linked to respiratory infections, particularly in people with cystic fibrosis, which lead to persistent lung infections and impaired lung function. Biofilms inhibit recovery, entail invasive procedures, and lead to recurring infections in the context of chronic infections, urinary tract infections, chronic otitis media, and gastrointestinal infections [10].

Biofilm-mediated immune evasion

Bacterial biofilms use a number of strategies to avoid detection by the host immune system. Bacteria frequently lower their metabolic activity within the biofilm making less vulnerable to immune cells that target actively growing microorganisms [4]. Furthermore, in order to avoid detection by the host immune system, biofilms can undergo antigenic variation and release chemicals that suppress immune signaling and activity. Several ways can be used to target these processes for therapeutic intervention. Using enzymes or biofilm-disrupting chemicals to disrupt the biofilm matrix can expose bacteria to immune cells [11]. Immunomodulatory treatments can improve clearance by modifying the immune response. Phage treatment, which employs viruses to target bacteria within biofilms has the potential to circumvent biofilm defense [12].

Biofilms in medical devices

Bacterial biofilms play an important role in the development and spread of infectious diseases through medical devices. Bacteria can stick to the surfaces of medical devices such as catheters, and prosthetic joints, and develop biofilms while inserted into the body. The bacteria are protected by biofilms, which operate as a barrier against the host immune system and medications [19].

To prevent and manage infections associated with medical devices following measures can be taken (Table 2).

**Table 2:** Preventive measures

Preventive measures	Description
Strict Hygiene Antimicrobial-Coated	Maintaining a sterile atmosphere and practising proper hand hygiene are crucial in preventing early contamination [21].
Equipment and Aseptic Procedures	Antimicrobial coatings are now available on some medical equipment, which restrict bacterial attachment and biofilm formation [22] .
Catheter Care Bundles	By addressing issues such as appropriate catheter placement, maintenance, and prompt removal, catheter care bundles can lower the risk of infection [23].
Early detection	Monitoring for symptoms of infection, such as fever or local inflammation, can lead to early discovery and action, potentially lowering the severity of the infection [24].
Antibiotic Prophylaxis	To reduce risk of infection, healthcare providers may give prophylactic antibiotics prior to device placement in some circumstances [25].

Impact of bacterial biofilms on infection recovery

Bacterial biofilm development is harmful in infection recovery and tissue repair processes. Biofilms generate a hostile environment marked by chronic inflammation, hampered tissue regeneration and protected recovery. Biofilm bacteria cause damage to enzymes and poisons that harm host tissues and slow the recovery process [4]. The development of innovative infection treatment techniques precisely targets biofilm-related problems [17]. Innovative infection dressings with biofilm-disrupting properties, topical antimicrobials tailored to penetrate biofilms, growth factors to stimulate angiogenesis, immune-modulating therapies to boost host defense and advanced infection monitoring techniques for early detection and intervention are among the strategies being used [26].

Challenges in diagnosing bacterial biofilms

Biofilms are frequently neglected by conventional culture-based approaches, which are better suited to free-floating microorganisms [4]. This can result in delayed or erroneous diagnoses and as a result ineffective treatment. Furthermore, the mild and diverse clinical presentation of biofilm-associated infection can impede timely identification. Molecular techniques are polymerase chain reaction and metagenomics which allow for the identification of biofilm-forming bacteria and their unique genetic markers [27]. Fluorescence microscopy with particular stains can visualize biofilm architecture in clinical samples providing direct confirmation of their presence. Techniques such as matrix-assisted laser ionization time-of-flight mass spectrometry allows for the quick and exact detection of biofilm-associated organisms. Furthermore, sophisticated imaging techniques like confocal laser scanning microscopy and nuclear imaging enable for non-invasive visualization of biofilms in vivo [13].

Enhancing antibiotic efficacy against biofilms

The presence of bacterial biofilms significantly reduces the efficacy of antimicrobial medicines, offering a significant difficulty in treating biofilm-associated infections [4]. Biofilms form a protective fortress around bacteria making it extremely difficult for antibiotics to enter and exercise their effects [28]. Novel therapeutic techniques are being investigated in order to overcome these obstacles and improve drug penetration and activity within biofilms [29]. Among these are the use of multiple antibiotics in combination therapies, use of biofilm-disrupting agents that are enzymes and compounds to break down the matrix, the use of nanoparticles or drug delivery systems to improve antibiotic release and penetration, the development of anti-biofilm compounds that target the biofilm formation process itself. These novel approaches aim to address biofilms' distinct resistance mechanisms and provide hope for more effective treatment of biofilm-associated infections [15].

Factors influencing bacterial biofilm formation

A number of factors regulate the creation and persistence of bacterial biofilms in various anatomical regions [30]. Temperature, pH, oxygen levels, and nutrition availability are examples of local environmental factors that might encourage or impede biofilm formation [19]. Biofilm production and persistence are further influenced by host factors such as immunological response, underlying health problems, and genetics [14]. Understanding these variables helps to inform focused prevention and treatment efforts, allowing for specialized methods that address the distinct problems provided by biofilms at different anatomical regions [31]. This information can be used to develop more effective ways of combating biofilm-related infection and improving patient outcomes [28].

Interactions within bacterial biofilms

Interspecies interactions within bacterial biofilms have important impact on pathogenicity and virulence. These interactions can increase the aggregate virulence of biofilms, making them more infectious [4]. Synergistic interactions between diverse bacterial species within a biofilm can result in the synthesis of virulence factors such as toxins and enzymes not normally produced by individual species [32]. These components can cause tissue damage, dampen the immune response, and add to the biofilm's overall pathogenicity [33]. Traditional techniques that target single species may be ineffective against biofilms because numerous species collaborate to produce a hostile environment. Instead, treatment strategies should take into account the complex relationships between species aiming on disrupting these interactions and targeting shared virulence pathways [29].

Biofilms and chronic inflammatory diseases

Bacterial biofilms can provide a persistent and resilient environment for dangerous bacteria within the body which contributes to the development of chronic inflammatory diseases. Biofilms can avoid detection by the immune system. The extracellular matrix that surrounds the bacteria protects it from immune cells making it harder for the body to rid itself of the infection. This results in a prolonged inflammatory response as the immune system seeks to combat the infection on an ongoing basis resulting in chronic inflammation [16]. Biofilms are frequently antibiotic-resistant. Antibiotic penetration can be limited by the structure of the biofilm, and some bacteria within the biofilm enter a latent state, making them less sensitive to medications. As the infection persists, antibiotic resistance contributes to chronic inflammation [34].

## Conclusions

Biofilms are the basic source of causing acute and chronic diseases. Biofilms occur due to improper management of medical devices; there should be an enhanced multidisciplinary approach involving microbiologist clinicians and researchers. They are commonly associated with medical devices and can lead to persistent infections. Improved diagnostics and a deeper understanding of biofilm formation factors are also essential, enhancing the knowledge of biofilm-related diseases and causing chronic infection and their following treatment. Due to biofilms, many diseases have been taking place, so we should be aware of it and take preventive measures related to it. Many challenges have been overcome, and delays in the diagnosing process have resulted in ineffective treatment. Biofilms also play a major role in the complex microbial communities, shielded by an extracellular matrix, exhibiting increased antibiotic resistance, immune evasion, and tissue damage. Overcoming these challenges requires innovative approaches, such as disrupting the biofilm matrix, utilizing combination therapies, and exploring phage therapy.
